# Patient-Specific CT-Based Instrumentation versus Conventional Instrumentation in Total Knee Arthroplasty: A Prospective Randomized Controlled Study on Clinical Outcomes and In-Hospital Data

**DOI:** 10.1155/2015/165908

**Published:** 2015-08-02

**Authors:** Andrzej Kotela, Jacek Lorkowski, Marek Kucharzewski, Magdalena Wilk-Frańczuk, Zbigniew Śliwiński, Bogusław Frańczuk, Paweł Łęgosz, Ireneusz Kotela

**Affiliations:** ^1^Department of Orthopedic Surgery and Traumatology, Central Research Hospital of Ministry of Interior, Wołoska 137, 02-507 Warsaw, Poland; ^2^Department of Orthopaedics and Traumatology of Musculoskeletal System, 1st Faculty of Medicine, Medical University of Warsaw, ul. Lindleya 4, 02-005 Warsaw, Poland; ^3^School of Medicine with the Division of Dentistry in Zabrze, Chair and Department of Descriptive and Topographic Anatomy, Medical University of Silesia, ul. Jordana 19, 41-808 Zabrze, Poland; ^4^Department of Kinesiotherapy and Manual Therapy, Faculty of Health Sciences, Vincent Pol University in Lublin, ul. Choiny 2, 20-816 Lublin, Poland; ^5^Head of Institute of Physiotherapy, The Jan Kochanowski University of Humanities and Sciences, al. IX Wieków 19, 25-317 Kielce, Poland; ^6^Department of Physiotherapy, Faculty of Health and Medicine Science, Andrzej Frycz Modrzewski Cracow University, ul. Gustawa Herling Grudzińskiego 1, 30-705 Kraków, Poland; ^7^Department of Physiotherapy, The Jan Kochanowski University of Humanities and Sciences, al. IX Wieków 19, 25-317 Kielce, Poland

## Abstract

Total knee arthroplasty (TKA) is a frequently performed procedure in orthopaedic surgery. Recently, patient-specific instrumentation was introduced to facilitate correct positioning of implants. The aim of this study was to compare the early clinical results of TKA performed with patient-specific CT-based instrumentation and conventional technique. A prospective, randomized controlled trial on 112 patients was performed between January 2011 and December 2011. A group of 112 patients who met the inclusion and exclusion criteria were enrolled in this study and randomly assigned to an experimental or control group. The experimental group comprised 52 patients who received the Signature CT-based implant positioning system, and the control group consisted of 60 patients with conventional instrumentation. Clinical outcomes were evaluated with the KSS scale, WOMAC scale, and VAS scales to assess knee pain severity and patient satisfaction with the surgery. Specified in-hospital data were recorded. Patients were followed up for 12 months. At one year after surgery, there were no statistically significant differences between groups with respect to clinical outcomes and in-hospital data, including operative time, blood loss, hospital length of stay, intraoperative observations, and postoperative complications. Further high-quality investigations of various patient-specific systems and longer follow-up may be helpful in assessing their utility for TKA.

## 1. Introduction

Total knee arthroplasty (TKA) is a widely recognized method of treatment for advanced osteoarthritis of the knee joint. It has proven to be efficient for eliminating progressive pain and restoring normal function of the operated joint. In most cases, TKA allows the patients to return to normal life activities and improves their quality of life significantly [1, 2].

Many studies aimed at increasing the accuracy of knee implant positioning and implant life expectancy have been recently conducted. The number of different types of prostheses on the market keeps growing, with increasingly newer tool sets designed to facilitate correct implantation. One particular modern solution for TKA involves systems where intraoperative navigation is based on single-use guides fashioned specifically for the patient. The main idea behind patient-specific instrumentation (PSI) is that TKA procedures can be individualized, with benefits including precise realignment of the normal mechanical axis of the operated lower limb, minimized resection of bone, reduced surgical times, simplified instrumentation, reduced peri- and postoperative blood loss, no femoral medullary cavity reaming, and reduced rate of thromboembolic complications [3–8].

As shown previously by other authors, improved accuracy in prosthetic alignment correlates with enhanced knee function and patient quality of life. According to Victor et al. [9], coronal plane outliers may lead to inferior functional outcomes, earlier loosening, and polyethylene wear [10–14]. Internal rotation of the femoral component is associated with pain, stiffness, and instability [15–20], whereas excessive external rotation of the femoral component can lead to symptomatic flexion instability, increased shear forces on the patella, and medial compartment overload in flexion [21–23]. Finally, excessive tibial slope in the sagittal plane leads to postimpingement and flexion instability in posterior stabilized designs [24].

To date, the number of papers addressing the clinical of PSI is very limited. Thus, the goals of this study were to assess and compare CT-based PSI to conventional TKA technique with regard to early clinical results and perioperative outcomes. Intraoperative observations, including changes from the planned implant to the definitively implanted component size, were also evaluated.

## 2. Material and Methods

**Figure 1:**
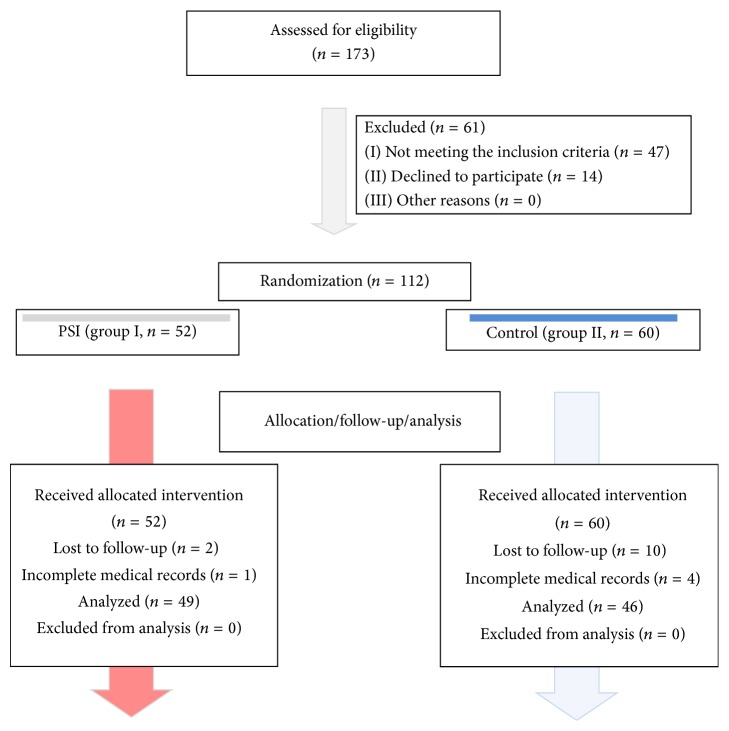
A flowchart illustrating the study design.

A prospective, randomized controlled trial (RCT) was conducted at a single institution from January 2011 to December 2011. There were 173 patients evaluated for the study, and 112 consecutive patients scheduled to undergo TKA were included in the study. 17 patients were excluded for the reasons listed below. The study was performed in accordance with its study protocol, which was approved by the ethical review board. Informed consent was provided by all patients. A flowchart of the study is shown in Figure 1.

Inclusion/exclusion criteria are as follows.

Inclusion criteria: consider the following:

- primary end-stage knee osteoarthritis,
- primary, uncemented TKA,
- implant: Vanguard (Biomet), cruciate retaining,
- age > 18 years old,
- provision of written informed consent.

Exclusion criteria: consider the following:

- nonosteoarthritic conditions as reason for operation (e.g., rheumatoid arthritis),
- comorbidities that might affect bone quality (e.g., osteoporosis),
- a history of previous surgery or trauma to the affected knee within 12 months preceding the study,
- cognitive disorders resulting in suboptimal cooperation of the patient,
- bilateral TKA,
- failure of the patient to present for a follow-up examination,
- incomplete medical records.

The participants were divided into two groups using a simple randomization procedure. Group I consisted of 52 patients who underwent TKA performed with the usage of patient-specific CT-based instrumentation (Signature Personalized Patient Care System; Biomet Inc., Warsaw, IN, USA). Group II consisted of 60 patients operated with conventional instrumentation. In all cases, the same type of uncemented, cruciate-retaining prostheses was implanted (Vanguard Complete Knee System; Biomet Inc., Warsaw, IN, USA), without resurfacing of the patella. All operations were performed by a single senior surgeon. The study group consisted of 66 (69.4%) women and 29 (30.6%) men. The average age was 67.3 years old (range: 41–80). Patients over 60 years old comprised 87% of the study group. The mean body mass index (BMI) was 29.8 kg/m², with 47.3% obese patients (BMI > 30 kg/m²) and no underweight patients. There were no statistically significant differences in patient demographics, clinical condition, and radiographic data prior to surgery (Table 1).

The process by which the Signature instrumentation is produced and is used has been described precisely in our previous report [7] and studies of other authors [3–5, 25–27]. All patients from Group II were operated on with traditional, jig-based instrumentation; extramedullary instruments were used for the tibial component, and intramedullary method was used for the femoral side. All patients in both groups received prophylactic intravenous antibiotics, and a medial parapatellar arthrotomy with the use of a tourniquet was used until the prostheses were implanted. The sizes of components planned based on computer aided surgical planning were compared to the sizes of the actual components implanted. At the end of surgery, two Redon drains were inserted into the deep joint space and were then removed on the second postoperative day, and blood reinfusion systems were not used. The volume of blood collected in Redon drains during the first two postoperative days was recorded for each patient and the tourniquet time was defined as incision until implantation of the prosthesis with final evaluation of its position. Blood was transfused in the postoperative period in all patients with hemoglobin levels less than 8 g/dL. The number of blood transfusions, period of hospitalization, and complications were also recorded. Deep vein thrombosis prophylaxis was the same in both groups [28].

Postoperative management was identical for both groups. Rehabilitation commenced on the first postoperative day. At discharge, patients were able to perform at least 90° flexion of the knee and moved about efficiently using elbow crutches. Follow-up routinely took place at 6 weeks and 3, 6, and 12 months after surgery.

Clinical outcomes were evaluated with the Knee Society Score (KSS), 5-point Likert scale version of Western Ontario and McMaster Osteoarthritis Index (WOMAC) scale, Visual Analogue Scale (VAS) to assess knee pain severity, and VAS scale to assess patient satisfaction with the surgery. Knee range of motion and knee axis were measured with a goniometer. Patients were examined before surgery and at 12 months after implantation of the knee prosthesis.

Statistical analysis (comparisons of groups) was performed using Fisher's exact test for qualitative variables and Mann-Whitney's *U*-test for quantitative variables (SAS, Inc., Chicago, Ill). Spearman's rank correlation coefficient was calculated to compare the planned implant to the actual implant inserted. A value of *p* < 0.05 was considered statistically significant.

## 3. Results

One year after surgery, preoperative and postoperative clinical outcomes significantly improved in both groups (Table 2). The preoperative scores were similar between groups, and outcomes obtained 12 months after surgery did not reveal any statistically significant differences between the groups in any of the scales (Table 2). Of note, the KSS and WOMAC scores were slightly better in Group II (170.2 ± 22.2 and 16.3 ± 14.7, resp.), compared to Group I (156.6 ± 33.2 and 17.1 ± 15.3, resp.). Group I had slightly better VAS pain scores (17.0 ± 16.7), although the difference compared to Group II was not significantly different (19.3 ± 17.2, *p* = 0.48). The overall mean VAS satisfaction score was 91.1 ± 11.3, and there was no significant difference between Group I (91.5 ± 11.1) and Group II (90.6 ± 11.4).

There were no differences in perioperative factors between groups. The mean tourniquet time was 34.0 ± 9.0 minutes in Group I (range: 20.0–72.0) and 28.5 ± 5.2 minutes in Group II (range: 21.0–52.0). The mean postoperative blood loss was 850 ± 450.5 mL in Group I and 1000 ± 502.1 mL in Group II. In the postoperative period, 6 patients (12.3%) from Group I required 1 unit of red blood cell (RBC) transfusion. In Group II, 5 patients (10.9%) required a RBC transfusion, of which 4 patients received 1 unit, and 1 patient required 3 units. The mean length of stay was 9.0 ± 3.8 days in Group I (range: 6–35) and 9.0 ± 1.5 days in Group II (range: 7–16). The longest period of hospitalization recorded in the study was 35 days, as a patient in Group I had a superficial postoperative wound infection and hospital-acquired pneumonia. There were no statistically significant differences between the groups in the duration of the surgery, postoperative blood loss, and length of hospitalization (Table 3).

No intraoperative complications were recorded. In Group I, there was one case of impaired wound healing from a superficial infection with suppurative discharge and marginal necrosis, and wound revision and debridement were performed on the tenth postoperative day. The patient was also diagnosed with hospital-acquired pneumonia in the second week following surgery. In Group II, two cases of postoperative intra-articular hematoma with significant swelling of the operated joint with delayed wound healing were recorded. Anticoagulation was held and these patients were treated nonoperatively.

Joint contracture requiring further arthroscopic treatment (lysis of adhesions) was diagnosed in one patient from Group I and two patients from Group II at one year after surgery. None of the patients analyzed in the study required revision arthroplasty procedures and no thromboembolic complications were recorded during 12 months following surgery.

No conversion to traditional instrumentation was reported in Group I. The sizes of the femoral component planned based on computer-aided preoperative planning were consistent with the sizes of the components that were implanted in 44/49 (89.8%) cases. The femoral component implanted was one size larger in one patient and one size smaller in four patients. The planned sizes of the implants and sizes of the tibial components implanted were consistent in 37/49 (75.5%) of patients. The implanted tibial component was one size larger in five cases and two sizes larger in one case. In six cases, the tibial component implanted was smaller by one size. The planned thickness of the polyethylene insert was 10 mm for all patients. Eight patients were implanted with a 12 mm polyethylene insert and two patients received 14 mm inserts. The planned size of the insert was consistent with the implanted insert in 39/49 (79.6%) of patients. The rate of consistency between the planned sizes of the component and the implanted sizes of the component was 0.99 (*p* < 0.0001) for the femoral component and 0.94 (*p* < 0.0001) for the tibial component. Femoral, tibial, and polyethylene insert sizes were similar in both groups (Table 3).

## 4. Discussion

Treatment of osteoarthritis varies depending on how advanced the disease is and what are the symptoms. An effective method for evaluation of a treatment process should take patients' outcomes into account and study parameters that are most important to patients. Thus, the purpose of our study was to evaluate the clinical outcome comparing PSI to conventional instrumentation, as eliminating pain and restoring correct knee function to allow patients to perform activities of daily living without assistance are the key goal of TKA from the patient's point of view.

To our knowledge, there are only 3 studies designed to evaluate the clinical outcomes of PSI in literature. Abdel et al. [29] performed a RCT to assess whether MRI-based PSI improves patient-reported outcomes and gait parameters at 3 months after surgery. They found no difference between PSI and conventional instrumentation groups in terms of KSS, Knee Injury and Osteoarthritis Outcome Score (KOOS), SF-12, and 3D gait parameters. Woolson et al. [30] conducted an RCT comparing outcomes of patients treated with custom and traditional instrumentation and found that there were no significant differences between both groups with regard to KSS at six-month follow-up examination. Finally, Pietsch et al. [31] performed an RCT to evaluate blood loss and early clinical outcomes in 40 patients undergoing minimally invasive TKA using custom-fit MRI-based instrumentation and 40 TKA patients with traditional instrumentation. There were no differences in terms of KSS, knee flexion, knee swelling, and pain at 3-month follow-up.

In our series, all patients exhibited a substantial and statistically significant improvement in KSS, WOMAC, and patient quality of life. There were also full or significant resolution of pain symptoms, based on the VAS pain scale, and high patient satisfaction from treatment. However, there were no significant differences at 12-month follow-up between PSI and conventional instrumentation for KSS, WOMAC, VAS pain, and VAS satisfaction. Our results are comparable to those of other authors and confirm their findings. In our opinion, this affirms that the study followed a correct procedure and adds credibility to our findings.

Since PSI does not require femoral medullary cavity reaming, the use of this method has previously been shown to reduce peri- and postoperative blood loss and lower the rate of thromboembolic complications. Increased rates of pulmonary embolism in cases where intramedullary guides were used were reported by Lotke and Ecker [11], Kolettis et al. [32], and Caillouette and Anzel [33]. The results of the study performed by Kalairajah et al. [34] suggested that computer-assisted TKA decreases systemic emboli significantly, when compared with conventional jig-based surgery.

In our study, we did not show a statistically significant difference in postoperative blood loss between the two groups; nevertheless, the estimated blood loss was lower in Group I by 150 mL. It should be noted that the hole in the femur from intramedullary reaming was filled with autologous bone graft in all Group II patients. Similarly, in the study by Roh et al. [5], blood loss estimated via postoperative drain flow did not differ between groups (783.7 mL in the CT-based Signature PSI group versus 843.8 mL in the conventional instrument group). Boonen et al. [35] reported significantly lower intraoperative blood loss in the group of patients operated on with the MRI-based Signature system (193.2 mL) compared to those who underwent conventional method (297.9 mL, *p* < 0.001). In contrast, Stronach et al. [27] analyzed intraoperative blood loss and found no difference between the MRI-based Signature (114 mL) and standard instrumentation groups (107 mL).

High operative blood loss was managed with transfusions of homologous blood from a blood bank. In our series, 11 patients required at least one unit of RBC transfusion. As reported by various authors, the percentage of patients after TKA who require a RBC transfusion ranges from 33 to 75%, and one unit of RBC is usually sufficient [36–38]. Cut bone structures are a significant source of postoperative bleeding, which is particularly important in uncemented arthroplasty. Since most studies refer to cemented arthroplasty, the percentage of patients in our study who required RBC transfusions should be considered as relatively low. The use of CT-based Signature PSI did not noticeably decrease the frequency of RBC transfusions.

TKA, as with any other surgical intervention, bears a risk of various complications. The incidence of complications in postoperative wound healing in patients after TKA ranges from 1 to 10% [39, 40]. In our study, we reported three such cases (6.1%), including two cases of intra-articular hematoma in conventional instrumentation patients and one case of a superficial wound infection in the PSI group with hospital-acquired pneumonia and a history of other comorbdities (cirrhosis, chronic atrial fibrillation, chronic heart failure, chronic obstructive pulmonary disease, diabetes, and hypothyroidism). Three patients did not achieve active flexion of the operated joint of over 90° at follow-up. Since arthrofibrosis prevents correct rehabilitation and patient's functioning after surgery, all patients with postoperative knee joint rigidity were treated by arthroscopic lysis of the adhesions. The incidence of fixed flexion deformity following TKA can be as high as 17% [41]. In our study, the CT-based Signature PSI system did not influence the rate of complications after TKA.

In theory, simplification of instruments using PSI can result in reduced surgical duration. In a study by Boonen et al. [35], the duration of TKA was significantly shorter with the use of the MRI-based Signature guides than in conventional instrument TKA (44.7 ± 6.5 and 50.0 ± 10.6 min, resp.; *p* < 0.001). Bali et al. [42] also found a shorter skin-to skin time in 10 PSI cases compared retrospectively to contralateral TKA times. In contrast, Hamilton et al. [43] found that CT-based TruMatch PSI increased surgical time; the total surgical time was 4 minutes shorter for patients in the conventional group (54.7 min. versus 61.8 min., resp.; *p* < 0.01). In the study performed by Roh et al. [5], surgical time was also longer using the CT-based Signature PSI group than the conventional group (59.4 versus 46.6 min., resp.; *p* < 0.001). Nunley et al. [44] showed a marginal improvement and no statistically significant difference in tourniquet time for the MRI-based Signature system compared to conventional instrumentation (56 min. versus 61 min., resp.). Our findings confirm the results of the last study. In our material, the use of the Signature system resulted in a statistically insignificant increase in the duration of the surgery. It seems that the results obtained were different from those with longer durations due to inadequate fit of the PSI guides to the bone structures, as revealed in our previous report [7].

The possibility to evaluate the size of the prostheses before TKA is a theoretical advantage of PSI. This data could shorten the duration of the procedure, as well as reducing the quantity of implants delivered to the surgical unit each time. Currently, due to the possibility of significant discrepancies between the X-ray view and the patient's clinical condition, knee replacement procedures are only performed if a full range of prosthesis sizes are available. In our study, despite a high correlation between the planned sizes of prosthetic components and those actually implanted, differences were recorded in as many as 10.2%, 24.5%, and 20.4% for the femoral component, tibial component, and polyethylene insert, respectively. Since the surgical planning system in question does not allow the sizes of the implants to be determined unequivocally before surgery, its relevance for use seems to be limited. It should be emphasized that, in our series, every preoperative surgical plan was checked and, if necessary, changed by the surgeon himself. This may explain why our results are better than those reported by other authors and confirms the necessity of careful analysis of preoperative software planning. In the study by Stronach et al. [27], MRI-based Signature PSI predicted the implanted component size in only 23% of femurs and 47% of tibias. Lustig et al. [45] performed a study to evaluate the MRI-based Visionaire PSI. In their study, the planned size matched the surgeon's decision in 52% and 50% of cases for the femur and tibia, respectively. In the cases that did not match, the planned size was a size too small in 23.3% of the time for the tibia and 28.3% of the time for the femur. Koch et al. [46] assessed the results of 301 TKAs performed with CT-based McKnee© PSI. In their material, the planned size was changed in total of 10.8% of all 602 implanted components. The tibial component was changed in 53 cases (in 26 cases, they were upsized, and in 27 cases, they were a downsized), and the femoral component was changed in 12 cases (in 7 cases they were upsized and in 5 cases, they were downsized).

The current study has several strengths and limitations [7]. The strengths include the randomized and controlled design of the study. As it was the first series of CT-based PSI TKAs in Poland and CT-related costs hindered the execution of our study, the population size of both groups should be viewed as relatively high, especially in comparison with most of the other studies. Although all procedures were performed by one surgeon experienced in TKA with previous training in PSI Signature method, we acknowledge the potential influence of the learning curve on our findings. There was some room for bias in this study, as the first author was a member of the surgical team in all cases and cared for the patients after the procedure. There was a possibility that he would know the group they were assigned to during the clinical examination. Further limitations include using the estimated intraoperative blood loss, instead of actual blood loss, and the short follow-up period. Nevertheless, our follow-up period was similar to Pietsch et al. [31], and we wanted to assess the rate of functional recovery rather than the longevity of the prosthesis. Finally, only one CT-based PSI system was used in this study, and the results may not apply to other CT or MRI-based positioning guides.

There was no improvement in the precision of knee implant positioning and restoration of the correct mechanical axis of the operated limb using the Signature PSI system [7], but performing TKA still provided good clinical outcomes and high patient satisfaction rates in both groups. Our study showed no differences between CT-based Signature PSI and conventional instrumentation in both early clinical outcomes and perioperative data. Simplified instrumentation, no need to ream the femur medullary canal, as well as more accurate preparation for surgery via the possibility to analyze interactive, 3D software data, remains undeniable benefits of PSI. In our opinion, further high-quality investigations of various patient-specific systems and longer follow-up are necessary to assess the efficacy of this new approach before it is widely accepted.

## Figures and Tables

**Table 1:** Patient demographics and preoperative limb deformities: a between-group comparison.

	PSI group (Group I)	CI group (Group II)	*p* value
Gender: male/female	16/33	13/33	0.66
Operated side: left/right	21/28	20/26	0.99
Age [years]^#^	66.1 ± 8.4	68.6 ± 9.9	0.33
Weight [kg]^#^	82.0 ± 14.5	80.0 ± 15.1	0.52
Height [m]^#^	1.7 ± 0.1	1.6 ± 0.1	0.87
BMI [kg/m²]^#^	30.0 ± 4.6	29.6 ± 5.6	0.45
HKA [*n*°]^#*∗*^	188.2 ± 7.0	188.9 ± 7.1	0.29

PSI: patient-specific instrumentation, CI: conventional instrumentation, SD: standard deviation, BMI: body mass index, and HKA: hip-knee-ankle angle.

^#^Data presented as mean ± SD; ^*∗*^positive values indicate varus alignment.

**Table 2:** Clinical evaluation scales: preoperative and postoperative results.

	Group I (PSI)		Group II (CI)		*p* value
	Mean ± SD	Min.–Max.	Mean ± SD	Min.–Max.	
Preoperative results					
KSS knee	28.5 ± 13.6	2.0–60.0	33.7 ± 16.8	0.0–70.0	0.15
KSS function	39.4 ± 18.8	0.0–80.0	42.8 ± 18.4	0.0–80.0	0.32
KSS total	67.9 ± 24.3	6.0–120.0	76.5 ± 29.8	18.0–130.0	0.14
WOMAC pain	11.6 ± 2.9	4.0–18.0	12.0 ± 3.2	6.0–20.0	0.64
WOMAC stiffness	4.7 ± 1.9	0.0–8.0	4.0 ± 1.7	0.0–6.0	0.07
WOMAC function, daily living	43.2 ± 8.1	17.0–57.0	42.3 ± 10.9	18.0–62.0	0.79
WOMAC total	59.5 ± 11.5	23.0–77.0	58.3 ± 14.5	27.0–83.0	0.79
VAS pain	68.2 ± 15.6	40.0–100.0	68.4 ± 18.0	10.0–99.0	0.59
Postoperative results					
KSS knee	77.9 ± 16.9	38.0–100.0	83.5 ± 13.8	34.0–100.0	0.11
KSS function	78.8 ± 20.3	30.0–100.0	86.7 ± 14.5	50.0–100.0	0.07
KSS total	156.6 ± 33.2	78.0–200.0	170.2 ± 22.2	104.0–200.0	0.07
WOMAC pain	2.2 ± 2.9	0.0–10.0	2.0 ± 2.3	0.0–9.0	0.72
WOMAC stiffness	1.4 ± 1.7	0.0–6.0	1.6 ± 1.6	0.0–6.0	0.38
WOMAC function, daily living	13.4 ± 11.9	0.0–41.0	12.6 ± 11.5	0.0–36.0	0.79
WOMAC total	17.1 ± 15.3	0.0–55.0	16.3 ± 14.7	0.0–46.0	0.92
VAS pain	17.0 ± 16.7	0.0–73.0	19.3 ± 17.2	10.0–57.0	0.48
VAS satisfaction	91.5 ± 11.1	60.0–100.0	90.6 ± 11.4	64.0–100.0	0.74

PSI: patient-specific instrumentation, CI: conventional instrumentation, SD: standard deviation, KSS: Knee Society Score, WOMAC: The Western Ontario and McMaster Universities Arthritis Index, and VAS: Visual Analogue Scale.

**Table 3:** Comparison of perioperative data.

	Group I (PSI)		Group II (CI)		*p* value
	Mean ± SD	Min.–Max.	Mean ± SD	Min.–Max.	
Tourniquet time (min)	34.0 ± 9.0	20.0–72.0	28.5 ± 5.2	21.0–52.0	0.98
Blood loss (mL)	850.0 ± 450.5	0.0–1920.0	1000.0 ± 502.1	0.0–2100.0	0.18
Hospital stay (days)	9.0 ± 3.8	6–35	9.0 ± 1.5	7–16	0.15
Femoral size	67.6 ± 4.7	60–80	65.6 ± 3.2	60–75	0.08
Tibial size	73.1 ± 6.5	63–87	74.5 ± 3.5	63–83	0.21
Insert thickness	10.5 ± 1.3	10–14	10.3 ± 1.5	10–16	0.69

PSI: patient-specific instrumentation, CI: conventional instrumentation, and SD: standard deviation.
