# Corrigendum to “Patient-Specific CT-Based Instrumentation versus Conventional Instrumentation in Total Knee Arthroplasty: A Prospective Randomized Controlled Study on Clinical Outcomes and In-Hospital Data”

**DOI:** 10.1155/2018/6723963

**Published:** 2018-09-12

**Authors:** Andrzej Kotela, Jacek Lorkowski, Marek Kucharzewski, Magdalena Wilk-Frańczuk, Zbigniew Śliwiński, Bogusław Frańczuk, Paweł Łęgosz, Ireneusz Kotela

**Affiliations:** ^1^Department of Orthopedic Surgery and Traumatology, Central Research Hospital of Ministry of Interior, Wołoska 137, 02-507 Warsaw, Poland; ^2^Department of Orthopaedics and Traumatology of Musculoskeletal System, 1st Faculty of Medicine, Medical University of Warsaw, ul. Lindleya 4, 02-005 Warsaw, Poland; ^3^School of Medicine with the Division of Dentistry in Zabrze, Chair and Department of Descriptive and Topographic Anatomy, Medical University of Silesia, ul. Jordana 19, 41-808 Zabrze, Poland; ^4^Department of Kinesiotherapy and Manual Therapy, Faculty of Health Sciences, Vincent Pol University in Lublin, ul. Choiny 2, 20-816 Lublin, Poland; ^5^Head of Institute of Physiotherapy, The Jan Kochanowski University of Humanities and Sciences, al. IX Wieków 19, 25-317 Kielce, Poland; ^6^Department of Physiotherapy, Faculty of Health and Medicine Science, Andrzej Frycz Modrzewski Cracow University, ul. Gustawa Herling Grudzińskiego 1, 30-705 Kraków, Poland; ^7^Department of Physiotherapy, The Jan Kochanowski University of Humanities and Sciences, al. IX Wieków 19, 25-317 Kielce, Poland

In the article titled “Patient-Specific CT-Based Instrumentation versus Conventional Instrumentation in Total Knee Arthroplasty: A Prospective Randomized Controlled Study on Clinical Outcomes and In-Hospital Data” [1], an acknowledgment should be added as follows:

A very special gratitude goes to Professor Jarosław Deszczyński, Head of the Department of Orthopaedics and Rehabilitation, 2nd Faculty of Medicine, Medical University of Warsaw, for all help and sharing his pearls of wisdom.

We would also like to thank all our colleagues from Department of Orthopaedics and Rehabilitation, 2nd Faculty of Medicine, Medical University of Warsaw, who provided insight and expertise that assisted the research.

The results presented were part of the Ph.D. research partially supported by the Foundation for the Development of Fracture Stabilization Techniques “Dynastab”.
